# *Lippia graveolens* Essential Oil to Enhance the Effect of Imipenem against Axenic and Co-Cultures of *Pseudomonas aeruginosa* and *Acinetobacter baumannii*

**DOI:** 10.3390/antibiotics13050444

**Published:** 2024-05-14

**Authors:** Jorge O. Fimbres-García, Marcela Flores-Sauceda, Elsa Daniela Othón-Díaz, Alfonso García-Galaz, Melvin R. Tapia-Rodriguez, Brenda A. Silva-Espinoza, Andres Alvarez-Armenta, J. Fernando Ayala-Zavala

**Affiliations:** 1 Centro de Investigación en Alimentación y Desarrollo, A.C, Carretera Gustavo Enrique Astiazarán Rosas 46, Hermosillo 83304, Sonora, Mexico; jfimbres121@estudiantes.ciad.mx (J.O.F.-G.); agarciag@ciad.mx (A.G.-G.); bsilva@ciad.mx (B.A.S.-E.); 2 Departamento de Biotecnología y Ciencias Alimentarias, Instituto Tecnológico de Sonora, 5 de Febrero 818 Sur, Col. Centro, Ciudad Obregón 85000, Sonora, Mexico; melvin.tapia14987@potros.itson.edu.mx; 3 Departamento de Microbiología Molecular, Instituto de Biotecnología, Universidad Nacional Autónoma de México, Cuernavaca 62250, Morelos, Mexico; alvare3a@gmail.com

**Keywords:** *Lippia graveolens*, carvacrol, *A. baumannii*, *P. aeruginosa*, co-cultures

## Abstract

This research focuses on assessing the synergistic effects of Mexican oregano (*Lippia graveolens)* essential oil or carvacrol when combined with the antibiotic imipenem, aiming to reduce the pathogenic viability and virulence of *Acinetobacter baumannii* and *Pseudomonas aeruginosa*. The study highlighted the synergistic effect of combining *L. graveolens* essential oil or carvacrol with imipenem, significantly reducing the required doses for inhibiting bacterial growth. The combination treatments drastically lowered the necessary imipenem doses, highlighting a potent enhancement in efficacy against *A. baumannii* and *P. aeruginosa*. For example, the minimum inhibitory concentrations (MIC) for the essential oil/imipenem combinations were notably low, at 0.03/0.000023 mg/mL for *A. baumannii* and 0.0073/0.000023 mg/mL for *P. aeruginosa*. Similarly, the combinations significantly inhibited biofilm formation at lower concentrations than when the components were used individually, demonstrating the strategic advantage of this approach in combating antibiotic resistance. For OXA-51, imipenem showed a relatively stable interaction during 30 ns of dynamic simulation of their interaction, indicating changes (<2 nm) in ligand positioning during this period. Carvacrol exhibited similar fluctuations to imipenem, suggesting its potential inhibition efficacy, while thymol showed significant variability, particularly at >10 ns, suggesting potential instability. With IMP-1, imipenem also displayed very stable interactions during 38 ns and demonstrated notable movement and positioning changes within the active site, indicating a more dynamic interaction. In contrast, carvacrol and thymol maintained their position within the active site only ~20 and ~15 ns, respectively. These results highlight the effectiveness of combining *L. graveolens* essential oil and carvacrol with imipenem in tackling the difficult-to-treat pathogens *A. baumannii* and *P. aeruginosa*.

## 1. Introduction

Antibiotics are drugs used to treat infections caused by bacteria, which cause their elimination or limit their growth and multiplication [1]. However, the efficacy of these agents has been widely threatened due to a phenomenon known as antibiotic resistance. This refers to the set of mechanisms that bacteria employ to avoid the effect of antibiotics on them, with poor control of their use being one of the possible causes of microorganism mutation for developing this condition [2,3]. Antibiotic resistance represents a serious large-scale health problem, as it endangers global human development [3]; in 2019 alone, around 5 million people died from some disease related to resistant microorganisms [4], and it is estimated that this figure will increase to 10 million by 2050, accumulating a global cost of 100 trillion dollars [5]. In conjunction, the development of resistance has also limited the arsenal of available drugs [6]. Such is the case with carbapenem antibiotics, used for treating severe pathogens due to their broad activity and are considered last-line treatment. However, with the emergence of resistant strains, the efficacy of these treatments is compromised [7,8]. In this sense, a group of bacteria has been recognized as priority pathogens according to the WHO, due to their great capacity of resistance to antibiotics and have become of research interest. This is formed by the pathogens *Enterococcus faecium*, *Staphylococcus aureus*, *Klebsiella pneumoniae*, *Acinetobacter baumannii*, *Pseudomonas aeruginosa* and *Enterobacter* spp. [9,10]. Furthermore, these microorganisms are responsible for more than 50% of all clinical infections [11].

Within this group, two pathogens, *A. baumannii* and *P. aeruginosa*, stand out due to their markedly relevant resistance and mortality rates. *A. baumannii* is a bacterium that has stood out for its ability to withstand hostile environments and develop and exhibit resistance factors [12]. Between 2019 and 2020 alone, at the beginning of the COVID-19 pandemic, there was an increase in the number of infections caused by carbapenem-resistant *A. baumannii*, amounting to 79% more [13]. Furthermore, this pathogen’s overall prevalence of resistance to imipenem has been estimated at 53.8–76.8% [6], which may partly explain its high mortality rate, estimated to be as high as 68% [14]. Resistant *P. aeruginosa*-related infections also increased by 32% in 2020 [13], and this characteristic of resistance causes a high degree of mortality [15]. These rates vary widely, ranging from 32% to 64% in infections such as ventilator-associated pneumonia (VAP), sepsis in burn patients and bloodstream infections [16]. However, the situation can be exacerbated if the disease is caused by a multiresistant strain, in which case mortality can be as high as 70%. Accordingly, it has been estimated that the global rate of resistance to carbapenems by *P. aeruginosa* ranges from 10 to 50% [17].

In turn, antibiotic resistance can manifest itself through several mechanisms, the main ones being the alteration of membrane permeability, increased activity of efflux pumps, modification of the drug target site, biofilm formation and the presence of enzymes that degrade or modify antibiotics [18]. These last two mechanisms are factors of great interest because, on the one hand, biofilms provide the pathogen with the capacity to resist adverse environmental situations and protect it from external agents that may harm it, such as antibiotics, which makes its treatment more difficult [17,19]. On the other hand, resistance mediated by modifying enzymes, such as β-lactamases, represents a common cause of difficult-to-treat infections and provides bacteria with increased resistance to antibiotics [19]. For *A. baumannii*, β-lactamases of the OXA type are the predominant factor in carbapenem resistance in this bacterium [12], while in *P. aeruginosa*, the IMP-1 enzyme was the first metallo-β-lactamase (MBL) identified and associated with carbapenem resistance and one of the most widely distributed since its coding genes can spread between species [20].

Due to the increasing antibiotic resistance and diminishing effectiveness, clinical practice is searching for alternatives that can permeate this discouraging scenario. In this regard, the use of plant compounds has proven to be a good option as they exhibit characteristics such as low toxicity, accessibility, extensive medicinal use, and above all, a high antibacterial capacity related to their phytochemical composition [21,22,23]. A plant with great antibacterial activity is oregano in its different species (*Origanum vulgare* and *Lippia graveolens*), which has proven to be effective against microorganisms such as *Candida albicans*, methicillin-resistant *Staphylococcus aureus* (MRSA), *Salmonella enterica*, *P. aeruginosa* and *A. baumannii* [24,25,26,27]. This effectiveness is attributed to its main terpene compounds, carvacrol and thymol. In addition, one of the current trends is the combination of antibacterial agents, which has been shown to have enhanced antibacterial activity. For example, a study evaluated the combination of *O. vulgare* essential oil with polymyxin B against isolates of *A. baumannii* and observed a synergistic effect inhibiting its growth [28]. Similarly, another study revealed that when *A. baumannii* was cultured on a medium containing trans-cinnamaldehyde-β-lactam antibiotic and eugenol-β-lactam combinations, respectively, the bacterial growth was decreased compared to bacteria exposed to individual treatments [29]. Building upon these findings, this study explored the synergistic effects of *L. graveolens* essential oil and carvacrol combined with imipenem in axenic cultures and co-cultures, a perspective not extensively examined in previous research.

## 2. Results

### 2.1. Antibacterial Capacity of L. graveolens Essential Oil, Carvacrol and Imipenem against Axenic Culture and Co-Culture of P. aeruginosa and A. baumannii

#### 2.1.1. Formation of the Co-Culture

Two distinct morphological characteristics were identified in colonies grown in co-cultures (Figure S1), distinguishing the studied species; large, circular, white, raised, creamy colonies were identified as *A. baumannii*, while small, spotty, circular, white, poorly translucent colonies were established as *P. aeruginosa* [30,31,32,33,34]. It was determined that the 1:1000 ratio with 1 μL of *P. aeruginosa* and 999 μL of *A. baumannii* allowed optimal growth of both bacteria in the medium, so the following evaluations were performed using that established ratio (Figure S1C). This ratio showed a variation in growth of 1,127,495 CFU/mL for *A. baumannii* and 1,333,402 CFU/mL for *P. aeruginosa*. *A. baumannii* represented 48.89% of the total bacterial growth, while *P. aeruginosa* obtained 51.11%, compared with the initial inoculum, showing a downward readjustment in *A. baummannii* and an upward readjustment in *P. aeruginosa*.

Establishing a homogeneous co-culture of *A. baumannii* and *P. aeruginosa* offers several key advantages for this study. Firstly, a balanced co-culture could more accurately simulate polymicrobial infections, providing a more complex model for assessing treatment efficacy. Secondly, using this co-culture will help understand how treatments impact each species in a shared environment. Moreover, maintaining a balance in bacterial competition ensures that neither species outcompetes the other, which is important for evaluating the simultaneous effects of active compounds on both bacteria.

#### 2.1.2. MIC and MBC of *L. graveolens*, Carvacrol and Imipenem against Axenic and Co-Culture of *P. aeruginosa* and *A. baumannii*

Table 1 shows the MIC and MBC values for imipenem, *L. graveolens* essential oil, and carvacrol against *A. baumannii* and *P. aeruginosa*, both in axenic cultures and co-cultures. A notable observation is the increased dosage requirement for MBCs compared to MICs, indicating a higher concentration needed for bactericidal effects. For *A. baumannii*, the MBC of imipenem was twice as high as its MIC, while the essential oil and carvacrol required higher doses. Conversely, *P. aeruginosa* was more susceptible to the plant compounds, requiring only half the MIC necessary for *A. baumannii*.

Furthermore, in co-culture, the necessary doses for bactericidal action were elevated compared to individual cultures: imipenem required up to fourfold for *A. baumannii*, while the essential oil’s MIC was eightfold higher than that for *P. aeruginosa* alone. Carvacrol’s MIC needed to inhibit co-culture growth was double that for *A. baumannii*. These differential susceptibilities underscore the complexity of treating co-infections and highlight the importance of tailored antibacterial strategies. The results obtained at this stage made it possible to determine the inhibitory concentrations of each antibacterial against *A. baumannii* and *P. aeruginosa* bacteria grown individually and in co-culture. These concentrations were used in the antibacterial combination tests to establish their effect on the viability and virulence of the two bacteria under investigation.

### 2.2. Synergy of L. graveolens–Imipenem and Carvacrol–Imipenem against Axenic and Co-Cultures of P. aeruginosa and A. baumannii

Table 2 shows the fractional inhibitory concentration indices (FICIs) of the combinations of each plant compound with imipenem against bacterial axenic and co-cultures. This parameter was obtained from the sum of the Fractional Inhibitory Concentration (FIC) of each combined agent, obtained from the ratio between the MIC in the combination and the individual MIC (Table 1). For *A. baumannii*, using combinations of oregano essential oil–imipenem and carvacrol–imipenem at doses 21 times lower than the MIC of each agent was enough to inhibit its growth. In the case of *P. aeruginosa*, the same pattern of behavior was observed for the essential oil–imipenem combination, as 21.4 times less than the MIC of each agent was required. However, for the carvacrol–imipenem combination, 21.4 times less of the MIC of carvacrol and 10.7 times less of the MIC of imipenem was needed to inhibit bacterial growth.

The combinations of essential oil–imipenem and carvacrol–imipenem in co-culture showed a similar trend, since higher concentrations were required to inhibit co-culture growth compared to the axenic ones. It was necessary to use twice the concentration of oregano essential oil against co-cultures compared to that used in the combination against *A. baumannii* and eight times more than that required against *P. aeruginosa*. Meanwhile, when combined with oregano oil, the concentration of imipenem was two times higher in the co-culture than required to inhibit both bacteria’ axenic growth. The same behavior was observed for the carvacrol combination compared to the concentration needed in *A. baumannii*. Finally, twice the concentration of carvacrol was necessary for the co-culture for *A. baumannii* in the combination test. At the same time, in comparison with *P. aeruginosa*, it required four times the concentration in the co-culture system. However, it was evident that the combinations of treatments could considerably reduce the doses used for inhibition in both types of systems.

The FICIs of the lower concentrations were established between 0.0934 and 0.14041, indicating that the proposed combinations had a synergistic effect in inhibiting bacterial growth. The combination of antibacterial agents is a practice that has gained great relevance, supported by its effectiveness in bacterial control [35].

### 2.3. Individual and Combined L. graveolens Essential Oil, Carvacrol, and Imipenem against Axenic and Co-Cultured Biofilms of P. aeruginosa, A. baumannii on Endotracheal Tubes

**Figure 1:**
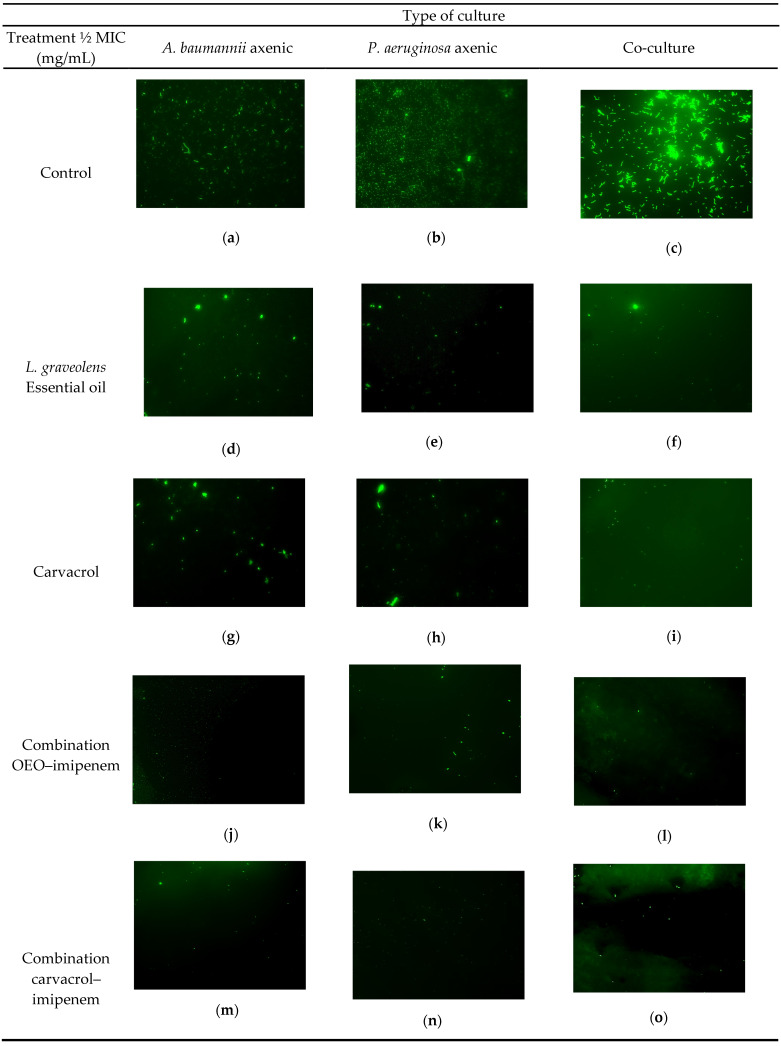
Fluorescence microscopy (200×) of biofilms of *A. baumannii*, *P. aeruginosa* and their co-culture on glass coverslips, incubated at 37 °C for 24 h in LB broth exposed to ½ MIC of the treatments essential oil of *L. graveolens* (EO), carvacrol and combinations with imipenem, stained with Syto 9 at 0.001%. (**a**) Control *A. baumannii*, (**b**) control *P. aeruginosa*, (**c**) co-culture control, (**d**) biofilm of *A. baumannii* exposed to 0.312 mg/mL EO, (**e**) biofilm of *P. aeruginosa* exposed to 0.078 mg/mL of EO, (**f**) co-culture biofilm exposed to 0.625 mg/mL of EO, (**g**) biofilm of *A. baumannii* exposed to 0.075 mg/mL of carvacrol, (**h**) biofilm of *P. aeruginosa* exposed to 0.0375 mg/mL carvacrol, (**i**) co-culture biofilm exposed to 0.15 mg/mL carvacrol, (**j**) *A. baumannii* biofilm exposed to EO–imipenem combination of 0.015/1.17 × 10^−5^ mg/mL, (**k**) *P. aeruginosa* biofilm exposed to EO–imipenem combination of 3.65 × 10^−3^/1.17 × 10^−5^ mg/mL, (**l**) co-culture biofilm exposed to EO–imipenem combination of 0.03/2.34 × 10^−5^ mg/mL, (**m**) biofilm of *A. baumannii* exposed to the carvacrol–imipenem combination of 3.5 × 10^−3^/1.17 × 10^−5^ mg/mL, (**n**) biofilm of *P. aeruginosa* biofilm exposed to the carvacrol–imipenem combination of 1.75 × 10^−3^/2.34 × 10^−5^ mg/mL, (**o**) co-culture biofilm exposed to the carvacrol–imipenem combination of 0.007/2.34 × 10^−5^ mg/mL.

Table 3 and Figure 1 detail the inhibitory effects on biofilm formation for *A. baumannii, P. aeruginosa*, and their co-culture when treated with *L. graveolens* essential oil, carvacrol, imipenem, and their combinations. *P. aeruginosa* biofilms demanded higher concentrations of *L. graveolens* essential oil and imipenem for inhibition compared to *A. baumannii*. Specifically, *L. graveolens* essential oil required a 2.25-fold greater concentration and imipenem a 1.8-fold increase, while carvacrol needed the same concentration for both pathogens. On the contrary, the inhibitory concentration of *L. graveolens* essential oil in co-culture is comparable to that employed for *P. aeruginosa* axenic culture (3 and 2.7 mg/mL, respectively). However, in the case of carvacrol, it is necessary to use concentrations 10 times greater than those of axenic cultures. Notably, imipenem’s effect on biofilm formation in the co-culture system is more effective than in axenic cultures. This highlights the potential of combined treatments for treating antibiotic-resistant bacteria.

Remarkably, the treatment combination exhibited a pronounced reduction in the doses needed to inhibit biofilm formation. For instance, combining 0.06 mg/mL of oregano oil with 2.35 × 10^−5^ mg/mL of imipenem reduced *A. baumannii* biofilm establishment, marking a 20- to 21-fold dose reduction compared to their individual applications. The carvacrol/imipenem combination also displayed this dose-lowering effect, requiring 3.3 to 3.5 times less concentration than when used individually. This synergistic effect extended to the treatment of co-cultures, where the *L. graveolens* oil/imipenem combination effectively inhibited biofilms at significantly reduced doses—five times less for the oil and nearly three times less for imipenem compared to their separate use. The carvacrol/imipenem combination maintained this trend, showcasing an equal concentration reduction factor.

Fluorescence microscopy images ratified these findings (Figure 1), revealing a substantial decrease in biofilm formation when treatments were applied, particularly with antimicrobial combinations. Notably, irrespective of the culture type, the concentrations necessary to inhibit biofilm formation exceeded those required to suppress planktonic bacterial growth. For instance, the dose of *L. graveolens* essential oil needed to reduce *A. baumannii* biofilm growth was double that for planktonic inhibition. In contrast, *P. aeruginosa* biofilms required an even higher, 17-fold increase in the essential oil concentration over the MIC. Similarly, the carvacrol dose increased eight-fold, and the imipenem dose nearly doubled to inhibit biofilm formation compared to their planktonic counterparts. In co-culture systems, biofilm inhibition called for a 2.4-fold increase in *L. graveolens* essential oil concentration and a 20-fold rise in the carvacrol dose, while imipenem’s biofilm inhibitory dose was half that of its planktonic MIC, underscoring the nuanced dynamics of biofilm response to treatment.

These results reflect the potent inhibitory effects of *L. graveolens* essential oil, carvacrol, and imipenem on the biofilm formation of *A. baumannii*, *P. aeruginosa*, and their co-culture, with the combinations revealing a remarkable synergy that significantly reduces the required doses.

### 2.4. In Silico Binding Affinity and Dynamic Stability of Molecular Complexes between OXA-51 and IMP-1 β-Lactamases from A. baumannii and P. aeruginosa with Carvacrol, Thymol and Imipenem

#### 2.4.1. Binding Affinity

Table 4 presents the binding affinities and interactions of carvacrol, thymol, and imipenem within the active sites of OXA-51 and IMP-1 β-lactamases from *A. baumannii* and *P. aeruginosa*. Imipenem, as the natural substrate of these enzymes, showed the strongest binding affinity, serving as the benchmark for comparison. Specifically, imipenem’s binding strength showed the highest negative affinity energy, indicating a robust and likely stable interaction, particularly with OXA-51 at −6.1 Kcal/mol and IMP-1 at −5.5 Kcal/mol. Carvacrol followed closely, especially with OXA-51, exhibiting marginally lower affinity energy, suggesting a slightly less strong but still significant interaction. While still effectively binding, thymol displayed the least affinity.

The interactions at the atomic level, particularly with key amino acids like Arg260 and Ser80 for OXA-51, suggested a dynamic interplay where imipenem forms stabilizing hydrogen bonds. At the same time, carvacrol engages through both hydrogen bonding and hydrophobic interactions with conserved residues. Notably, thymol’s interactions diverge, lacking the specific bonding with catalytic site residues, which may account for its lower binding affinity.

#### 2.4.2. Complexes Stability by Dynamic Simulation

**Figure 2:**
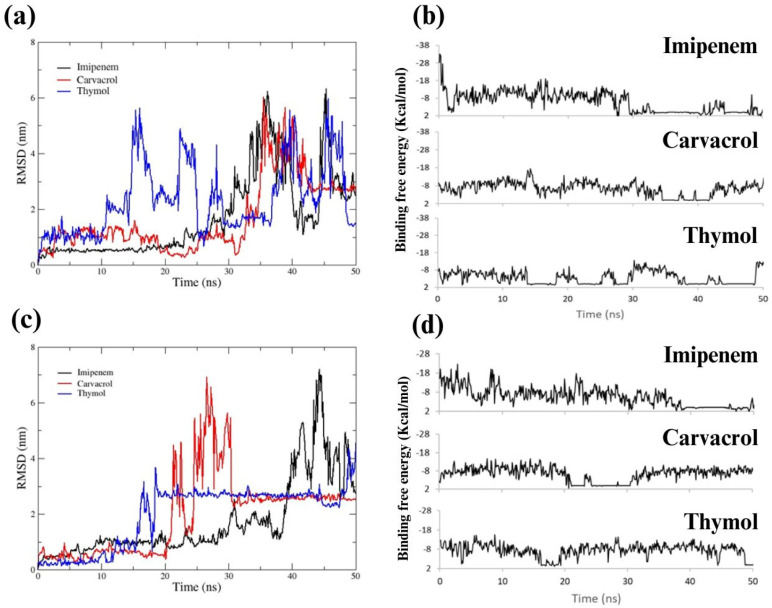
Deviation of the root mean square deviation of the atomic positions (RMSD) of imipenem, carvacrol and thymol and its binding free energy (Kcal/mol) with β-lactamase OXA-51 ((**a**) and (**b**), respectively) and IMP-1 (**c**,**d**). The behavior of imipenem is shown in black (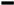), carvacrol in red (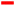) and thymol in blue (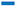).

The interactions between ligands and enzymes are dynamic phenomena; they evolve, influenced by the inherent stability of the initial complex. To capture the essence of this dynamism, the fluctuations of the ligand–enzyme complexes involving imipenem, carvacrol, and thymol with OXA-51 and IMP-1 β-lactamases were investigated. The resultant of ligand root mean square deviation (RMSD) profiles (Figure 2) represents the stability of the interactions between ligand and protein over time.

Figure 2 shows the RMSD and binding free energies of the ligands with β-lactamase OXA-51 (a and b) and IMP-1 (c and d). A stable RMSD curve, with minimal fluctuations, indicates a robust and enduring interaction, while significant variability suggests a more transient and potentially weaker binding. For OXA-51, imipenem demonstrates substantial stability (RMSD < 2 nm) during 30 ns of simulation, with notable deviations at later points, possibly signifying enzymatic processing. Carvacrol’s steadiness implies a durable interaction comparable to imipenem (~30 ns) with some alterations after 5 ns, while thymol shows pronounced variability after 10 ns, indicative that its interaction with OXA-51 is less favorable.

The ligand-OXA-51 RMSD patterns revealed that the imipenem dissociates around 40 ns, aligning with the expected enzymatic cleavage. Carvacrol remains anchored in the active site, suggesting a stable interaction throughout the simulation. Thymol, conversely, displays more pronounced movements, potentially affecting its binding stability. The insights gained from the RMSD patterns call for a more detailed examination of the crucial moments of interaction between the ligands and the OXA-51 enzyme, which are detailed in Supplementary Figures S2–S4. Similarly, the binding behavior of these ligands with IMP-1, depicted in Figure 2c, highlights the differences in variations of interactions between ligands over time. Notably, the RMSD trajectory for imipenem and carvacrol with IMP-1 remains relatively stable, with a discernible decrease in its stability for imipenem at 38 ns (likely indicative of hydrolytic process) and 20 ns, respectively. The detailed progression of these interactions over time can be further explored in Supplementary Figures S5–S7.

This analysis accentuates the importance of considering the dynamic nature of ligand–enzyme interactions in predicting the efficacy of antimicrobial agents. The observed binding behaviors, characterized by varying degrees of stability, provide a molecular basis for the synergistic effects seen in experimental biofilm inhibition assays.

## 3. Discussion

Establishing a co-culture between *A. baumannii* and *P. aeruginosa* is critical in modeling polymicrobial interactions, a common challenge in clinical infections such as cystic fibrosis and pneumonia. Our study’s ability to maintain a balanced co-culture mirrors recent research indicating the complexity of microbial communities where cross-feeding and biofilm production contribute to the synergy between co-infecting pathogens, often exacerbating disease severity and challenging antimicrobial treatments [36].

Our findings suggest a dynamic coexistence between *A. baumannii* and *P. aeruginosa*, potentially mediated by similar mechanisms of cross-protection as those reported in biofilm conditions, which can significantly alter antibiotic resistance profiles within microbial communities [37,38]. This cross-protection is particularly notable in our observations of the varying growth rates between the two bacteria, aligning with recent studies that have found that *A. baumannii* can protect other bacteria within biofilms, thereby enhancing resistance to antibiotics [39]. Additionally, the higher proportions of *P. aeruginosa* in our co-culture may be attributed to its virulence factors and the ability to produce compounds detrimental to neighboring microbes, as seen in other studies where *P. aeruginosa*’s toxic by-products lead to increased susceptibility and even lysis of other bacteria like *S. aureus* [40,41]. This aligns with the concept that polymicrobial interactions are not solely competitive but can also include protective and symbiotic relationships that facilitate coexistence and even shared resistance to environmental stresses.

The implications of these results extend to the understanding of in vivo infections, similar to observations made by Ramsey et al., where pathogen persistence was reliant on cross-feeding by a co-infecting pathogen with the exchange of by-products, such as ethanol and lactate [42]. This phenomenon between *A. baumannii* and *P. aeruginosa* could play a role in their co-persistence and impact on biofilm formation. The potential for shared siderophore utilization and cross-feeding between pathogens could necessitate a re-examination of antibiotic regimens to ensure that treatment is effective against the collective resistance mechanisms employed by co-infecting pathogens. This study advances the understanding of co-culture formation and stability and contributes to the broader narrative of microbial relations in polymicrobial interactions, highlighting the need for innovative treatment strategies that consider the dynamic and synergistic nature of these complex bacterial communities.

The establishment of co-culture between *A. baumannii* and *P. aeruginosa* marks an advancement in the simulation of polymicrobial infections. Dynamic adjustments observed in microbial populations during the study reflect the complexities created by species interaction. The strains examined demonstrated sensitivity to imipenem according to CLSI guidelines [43], suggesting its continued relevance in treating such infections.

The *Lippia graveolens* essential oil used in this study was characterized in detail in a previous study carried out by Rodriguez-Garcia et al. [44] by using gas chromatography–mass spectrometry, where carvacrol was identified as the predominant component (47.4%), accompanied by significant amounts of p-cymene (26.4%) and smaller fractions of thymol (3%), among others. The consistency in the major components such as carvacrol, p-cymene, and thymol aligns with findings from other studies [45]. Additionally, DMSO as a solvent was necessitated by the low aqueous solubility of the essential oil components, which might pose challenges for therapeutic applications.

MICs and MBCs for *L. graveolens* essential oil and carvacrol against axenic cultures align with previous investigations, including those with *O. vulgare* essential oil [27] [46,47,48]. This places our findings in an established scientific context and expands on the efficacy of treatment combinations against axenic and co-cultures. Notably, the differential doses required for *A. baumannii* compared to *P. aeruginosa* underscore the influence of specific virulence factors like the polysaccharide capsule in bacterial resistance mechanisms [49,50]. The study indicates that combining plant-derived compounds with imipenem may offer a promising advance for clinical therapy in the context of bacterial resistance. These combinations exploit distinct mechanisms of action—disruption of membrane integrity by essential oils and inhibition of cell wall synthesis by imipenem—potentially enhancing antibacterial outcomes [48,51,52,53,54,55,56,57,58,59]. Such strategies could contribute significantly to the fight against rising antibiotic resistance by varying the approaches to pathogen inhibition.

Synergistic effects observed in *L. graveolens*–imipenem and carvacrol–imipenem combinations across both axenic and mixed cultures highlight the potential of integrative therapies to augment the action of traditional antibiotics [28,60,61,62]. This is particularly pertinent considering the resistance mechanisms activated during co-culture conditions, including the upregulation of β-lactamase genes, which may undermine the efficacy of monotherapies [41,63]. The results suggest that therapeutic strategies should consider the collective action of antimicrobial agents within polymicrobial environments. This approach may lead to more effective clinical interventions, addressing the intricate interplay of bacterial species and their resistance profiles. The insights gained provide a foundation for further investigation into the interactive dynamics of *A. baumannii* and *P. aeruginosa*, with implications for evolving clinical treatments against these resistant pathogens.

Biofilm formation in bacteria like *P. aeruginosa* and *A. baumannii* can significantly contribute to their resistance to antibiotics [27,39,64,65]. Recent research has highlighted the complexity of biofilm-associated antibiotic resistance. For instance, a study by Elfaky et al. [66] explored the antibacterial potential of 6-gingerol against various bacteria, including *P. aeruginosa* and *A. baumannii*, focusing on its impact on biofilm formation. Similarly, Yunus et al. [67] investigated bacterial biofilm growth and perturbation, revealing insights into the biofilm formation processes of these pathogens. Moreover, research by Celebi et al. [68] suggested that vitamins could potentiate antibiotic effects against multidrug-resistant strains of these bacteria, with a notable impact on biofilm formation. Abdullah and Younis [69] aimed to understand the influence of antibiotic concentrations on biofilm production by *A. baumannii* and *P. aeruginosa*, suggesting a direct relationship between biofilm formation capabilities and antibiotic resistance. These findings indicate that biofilm formation serves as a barrier protecting bacterial communities from antibiotic penetration and as a microenvironment facilitating the transfer of resistance genes. Therefore, targeting biofilm formation and maturation processes presents a promising strategy for enhancing the efficacy of antibiotics against biofilm-associated infections by *P. aeruginosa* and *A. baumannii*.

Incorporating this understanding into the current discussion, the findings reflect that *L. graveolens* essential oil and carvacrol are effective against biofilm formation in *A. baumannii*, *P. aeruginosa* and their co-culture. Moreover, these plant compounds combined with imipenem not only inhibited biofilm formation but also reduced the effective doses of the antibacterials required, which is in line with the strategy to combat bacterial resistance by using natural compounds that can disrupt biofilm formation and quorum sensing mechanisms [39,52,70,71,72]. The combination treatments have shown potential for inhibiting biofilms by acting on different target sites and stages of biofilm development [27,73,74,75,76,77,78,79,80,81,82,83]. This multipronged approach, targeting both the bacterial cell wall and disrupting biofilm integrity, holds the potential for more effective treatment strategies, providing an innovative direction in the fight against resistant bacterial infections.

The binding affinities of phytochemicals to bacterial enzymes such as Penicillin Binding Proteins (PBPs) and Elongation Factors like EF-Tu have revealed inhibitory interactions important in the quest for novel anti-resistance strategies [84,85]. In particular, Kaempferol and Elatine have exhibited strong binding affinities, with Elatine demonstrating a significant docking score against PBP, suggesting an effective blockade of the bacterial drug targets [86]. Such molecular interactions are relevant as they may prevent the enzymatic degradation of antibiotics, thus enhancing their efficacy. This concept is underlined by studies where terpenes, such as carvacrol, have been shown to bind with notable affinity to microbial proteins. For instance, carvacrol’s interaction with the E protein of the Dengue Virus serotype 2, with an affinity energy suggesting significant inhibition of enzymatic activity, underscores the antiviral and antibacterial potential of terpenes [87]. Similarly, molecular docking of natural compounds with NDM-1, an MBL from *P. aeruginosa*, revealed compounds with binding energies indicative of potential inhibitory action [88].

These insights are particularly relevant to the current study, which explores the molecular interactions between hydrolytic enzymes like OXA-51 and IMP-1 and plant-derived terpenes. The findings suggest that terpenes may bind favorably to these enzymes, possibly blocking their active sites and thereby reducing the drug’s degradation, especially carvacrol, which was more stable in the complex. Such a mechanism could explain the synergistic effect when these terpenes are combined with antibiotics like imipenem. Therefore, incorporating plant compounds could represent a significant advance in counteracting antibiotic resistance and decreasing bacterial virulence.

The broader implications of these findings are profound. They not only contribute to a deeper understanding of the antimicrobial mechanisms of terpenes but also open avenues for the development of innovative therapeutic strategies against bacterial resistance. By inhibiting crucial enzymes involved in resistance pathways, these natural compounds could be harnessed to enhance the efficacy of existing antibiotics, offering a complementary approach in the global battle against resistant infections.

## 4. Materials and Methods

### 4.1. Conditions for Co-Culture

The methodology for establishing co-culture growth was adapted from the protocols proposed by Chan et al. [63] and Gao et al. [37]. The strains used were *A. baumannii* (ATCC^®^ 19606) and *P. aeruginosa* (ATCC^®^ 10145), which were initially and individually cultured in Luria Bertani broth (LB, Sigma Aldrich, Toluca, Mexico State, Mexico) for 18–24 h at 37 °C and subsequently each inoculum was adjusted to a concentration of 1 × 10^8^ CFU/mL in the logarithmic phase of growth. Subsequently, equal concentrations in volume ratios 1:2, 1:1000 and 1000:1 of each species were taken to combine and left incubating at 37 °C for 18–24 h. Finally, serial dilutions were made to decrease concentration by 10^−1^, 10^−3^, 10^−5^ and 10^−7^ and 100 μL of the co-culture was inoculated by mass seeding at a concentration of 1 × 10^8^ CFU/mL on LB agar plate in triplicate and incubated at 37 °C for 18–24 h. Both strains were detected and counted by visual inspection of the different colony morphologies (Figure S1).

### 4.2. MIC and MBC of L. graveolens, Carvacrol and Imipenem on P. aeruginosa and A. baumannii

The methodologies proposed by Bernal-Mercado [89], modified from CLSI, were used to determine the MIC and MIC of the essential oil of *L. graveolens* (Ore^®^ organic essential oil, Chihuahua, Mexico), carvacrol (Sigma Aldrich, Mexico State, Mexico) and imipenem (Sensimitina, Mexico, 500 mg/500 mg powder for solution). The various concentrations of carvacrol (0–2.25 mg/mL), *L. graveolens* essential oil (0–20 mg/mL) and imipenem (0–2 μg) were evaluated individually against *P. aeruginosa* and *A. baumannii* strains in their axenic and co-cultured form. The serial microdilution technique was used in these tests. For this, a microplate (Costar 96, Sigma Aldrich) was used where in each well, 5 μL of a culture adjusted to 1 × 10^8^ CFU/mL was inoculated to obtain a final concentration of 1 × 10^5^ CFU/mL; and 300 μL of broth containing each antibacterial was added at different concentrations, incubating at 37 °C for 24 h. The antibacterials were prepared in LB broth (Merck, Darmstadt, Germany) and 100 μL of DMSO (Merck) to achieve better oil solubility and carvacrol. Dilutions, where inhibition was observed, were identified, and 20 μL of each identified dilution was inoculated on plates with 20 mL of Mueller–Hinton agar free of the antimicrobial agent. The MIC was established as the lowest concentration of the treatments capable of completely inhibiting bacterial growth. On the other hand, MBC was defined as the lowest concentration of the antibacterials that caused loss of viability. These determinations were made in triplicate.

### 4.3. Effect of L. graveolens–Imipenem and Carvacrol–Imipenem Essential Oil Combinations on Axenic and Co-Cultures of P. aeruginosa and A. baumannii

The method proposed by Guo et al. [61] to analyze antibacterial combinations and by Canut-Blasco et al. [90] of the Spanish Society of Infectious Diseases and Clinical Microbiology was adapted and used. Different concentration fractions were established based on the MIC of each antibacterial agent against the cultures used. A 96-well microtiter plate (Fluostar Omega, BMG Labtech with 12 columns and 8 rows, Ortenberg, Germany) was used and the corresponding wells were inoculated with 100 μL of a bacterial suspension (1 × 10^8^ CFU/mL). In turn, carvacrol and the essential oil were distributed in each column, while the concentrations of imipenem were placed in each row. To each well with the adjusted inoculum, the required concentration of antibacterial was added to a final volume of 200 μL (50 μL of each antibacterial). The plates were incubated at 37 °C for 24 h, and the absence of growth was detected visually and confirmed by OD readings at 600 nm. The following formulas were used to calculate the fractional inhibitory concentration (FIC) of each antibacterial:

(1) $$FIC\;Plant\;Compounds\left(PC\right)=\frac{MIC\;of\;PC\;in\;combination\;with\;antibiotic}{MIC\;of\;PC}$$

(2) $$FIC\;Antibiotic=\frac{MIC\;of\;antibiotic\;\mathrm{in}\;combination\;with\;PC}{MIC\;of\;antibiotic}$$

where PC is *L. graveolens* oil or carvacrol, respectively. The following Equation (3) is used to obtain the fractional inhibitory concentration index (FICI) of the combination:

FICI = FIC PC + FIC antibiotic(3)

And it is interpreted as follows (∑FIC = FICI):

- ∑FIC ≤ 0.5: combination with a synergistic effect.
- ∑FIC > 0.5 ≤ 4: combination indifferent or no interaction.
- ∑FIC > 4: combination with antagonistic effect.

### 4.4. Impact of L. graveolens Essential Oil, Carvacrol and Their Combination with Imipenem on P. aeruginosa, A. baumannii and Their Co-Culture Biofilms on Endotracheal Tubes

Biofilms were formed in 1 cm long portions of an endotracheal tube (Laboratorios Jayor, S.A. de C.V, Mexico City, Mexico) (14 mm); these fragments were aseptically placed into test tubes containing 5 mL of LB broth. The essential oil was added to each tube at concentrations ranging from 0.15 to 7.5 mg/mL, mixed with 4% DMSO. For carvacrol, concentrations of 0.15–6 mg/mL were used [91], while for the plant compound–antibiotic combination, the results obtained from the checkerboard technique were taken as a basis. Subsequently, bacterial inoculum previously incubated for 19 h at 37 °C in LB broth was adjusted in the tubes to a final concentration of 1 × 106 CFU/mL and incubated for 24 h at 37 °C. After the time had elapsed, the endotracheal tube portions were removed from the medium, washed with distilled water and placed in 5 mL of saline to be subjected to ultrasound (42 KHz) for 5 min at 25 °C. Serial dilutions were performed with the resulting material to determine the number of bacteria adhered per unit area (Log CFU/cm^2^), for which they were inoculated on LB agar for 24 h at 37 °C. The minimum inhibitory concentration of biofilm formation (MICB) was established as the lowest concentration of the antibacterial agent that inhibits bacterial adhesion without affecting the viability of planktonic cells.

### 4.5. Fluorescence Microscopy of Treated Biofilms

The variations in the morphological constitution of the biofilms exposed to essential oil, carvacrol and their combinations with imipenem were visualized by fluorescence microscopy [89,91]. To achieve better visualization, 1 cm^2^ glass surfaces (coverslips) were used to form the biofilms. This material was placed in a sterile container with LB broth. A bacterial inoculum was first added by adjusting the concentration to 1 × 10^6^ CFU/mL, to incubate at 37 °C for 24 h. These biofilms developed while exposed to a concentration lower than the MBIC of each plant compound or its combination with the antibiotic. A biofilm formed without adding the antibacterial compounds was used as a reference. Once the proposed time had elapsed, the glass surfaces were washed with distilled water and stained with Syto 9 at 0.001% (Invitrogen, ThermoFisher, Waltham, MA, USA) for 30 min to visualize viable bacteria. The Axio Vert 1 fluorescence inverted microscope (Carl-Zeiss, Hebron, KY, USA) was used to observe the preparations with 40× objective Alexa Fluor filter.

### 4.6. Molecular Docking of OXA-51 and IMP-1 Enzymes from A. baumannii and P. aeruginosa with Carvacrol and Thymol

For this analysis, molecular docking was performed by contemplating the competitive landscapes to evaluate the probable interaction sites between the OXA-51 and IMP-1 proteins expressed in *A. baumannii* and *P. aeruginosa*, respectively, with carvacrol, imipenem and thymol. For this, the interactions of the receptor proteins OXA-51 (PDB 4ZDX) and IMP-1 (PDB 1DDK) proteins, with imipenem (PubChem 104838), carvacrol (PubChem 10364) and Thymol (PubChem 6989) were analyzed with the AutoDocvina program version 1.1.2., in the UCSF Chimera version 1.16 software as a visualizer. For this, the active sites were pointed out by the coordinates shown in Table S1 for each enzyme in “x”, “y” and “z”. These sites were determined based on key amino acids and their neighbors, considering previous crystallographic structures and their relevance in enzymatic activity [92,93].

With the above information, using the AutoDocvina program, the affinity energies (Kcal/mol) were estimated to determine the most favorable positions between the potential inhibitors and the enzymes in each interaction. Subsequently, Discovery Studio 2021 Client software was used to determine the interactions between the ligand and the enzyme and the amino acids and atoms participating in them. Finally, to analyze the behavior of the enzyme–ligand complex over time and the changes in its stability, a molecular dynamics simulation was performed using GROMACS software (version 2023.4) [94]. The topologies of ligands and proteins were obtained using the CHARMM-GUI server (https://www.charmm-gui.org/ (accessed on 28 November 2023)), using the CHARMM force field parameters. The rectangular box system was defined within 1 nm distance of the protein–ligand complex in each plane, solvated with TIP3P water model and neutralized with Na^+^ and Cl^−^ ions (0.15 M).

Furthermore, the system was equilibrated and minimized through two 200 ps simulations at 30 °C, one with NVT (constant number of particles, volume and temperature) and one with NPT (constant number of particles, pressure and temperature). Subsequently, the simulation analysis time of the prepared protein–ligand systems was set to 50 nanoseconds under 1 atm pressure and 30 °C conditions. The generated trajectory files were used to obtain the ligand RMSD (root mean square deviation) of the protein–ligand complex through the tools gmx rms using the Gromacs package. The corresponding plots were performed in qtgrace software (https://sourceforge.net/projects/qtgrace/ (accessed on 10 December 2023)).

### 4.7. Statistical Analysis

Statistical analyses were performed using descriptive statistics to evaluate the experimental data. For the establishment of the co-culture, colony-forming units per milliliter (CFU/mL) served as the response variable, with the inoculum ratio acting as the variable of interest. In assessing antibacterial activity, the response variables included MIC and MBC, with the variation factor being the differing concentrations of the antibacterial agents tested. In examining plant compound combinations with imipenem, the analysis focused on the interaction of various antibacterial concentrations, considering the FICI as the primary response variable. Furthermore, investigating the effects of carvacrol, *L. graveolens* essential oil, and their synergistic mixtures with imipenem on biofilm formation involved a similar approach, where the MBIC was the response variable measured against the gradient of antibacterial concentrations.

## 5. Conclusions

The study successfully established a co-culture model, providing valuable insights into the interactions and resistance mechanisms in polymicrobial environments, a critical aspect often encountered in infections. The synergistic effects observed between *L. graveolens* essential oil and imipenem highlight the potential of integrating natural compounds with conventional antibiotics to combat bacterial resistance more effectively. This combination showed enhanced efficacy against individual cultures and proved to be particularly effective in co-culture settings, suggesting a novel approach to managing infections involving these two pathogenic bacteria. Further, the findings emphasize the role of biofilm formation in antibiotic resistance and the ability of *L. graveolens* essential oil to disrupt these biofilms. The combination with imipenem exhibited promising results in inhibiting biofilm formation, pointing towards a strategic method to tackle one of the key factors in persistent bacterial infections. Future research avenues emerging from this study include a deeper exploration of the molecular interactions between *L. graveolens* essential oil and imipenem, and their combined impact on other bacterial species. In vivo analysis to assess the safety and effectiveness of this combination would be crucial to translate these findings into practical applications.

## Figures and Tables

**Table 1:** Minimum inhibitory concentrations (MICs) and bactericidal concentrations (MBCs) of imipenem, *L. graveolens* essential oil and carvacrol against *P. aeruginosa* and *A. baumannii* in the axenic and co-cultured form at 1:1000 ratio.

	*A. baumannii*		*P. aeruginosa*		Co-Culture	
Antibacterial (mg/mL)	MIC	MBC	MIC	MBC	MIC	MBC
Imipenem	5 × 10^−4^	1 × 10^−3^	5 × 10^−4^	>2 × 10^−3^	5 × 10^−4^	4 × 10^−3^
Essential oil of *L. graveolens*	0.625	>15	0.156	0.312	1.25	>15
Carvacrol	0.150	>2.25	0.075	0.150	0.30	>2.25

Number of replicates > 3.

**Table 2:** Minimum inhibitory concentrations (MICs) and fractional inhibitory concentration indices (FICIs) in the combinations of imipenem, *L. graveolens* essential oil and carvacrol against *P. aeruginosa* and *A. baumannii* in the axenic and co-culture form that resulted in synergistic effects.

Type of Culture	EO *L. graveolens * (MIC, mg/mL)	Imipenem (MIC, mg/mL)	Effect (FICI)	Carvacrol (MIC, mg/mL)	Imipenem (MIC, mg/mL)	Effect (FICI)
*A. baumannii* axenic	0.03	2.34 × 10^−5^	Synergy (0.09)	7 × 10^−3^	2.34 × 10^−5^	Synergy (0.09)
*P. aeruginosa* axenic	7.30 × 10^−3^	2.34 × 10^−5^	Synergy (0.09)	3.5 × 10^−3^	4.69 × 10^−5^	Synergy (0.14)
Co-culture	0.06	4.69 × 10^−5^	Synergy (0.14)	0.014	4.69 × 10^−5^	Synergy (0.14)

Number of replicates > 3. EO: essential oil.

**Table 3:** Minimum biofilm inhibitory concentrations (MBICs) of *L. graveolens* essential oil, carvacrol, imipenem and their combinations on *A. baumannii*, *P. aeruginosa* and their co-culture.

Type of Culture	Minimum Biofilm Inhibitory Concentrations (MBICs, mg/mL)				
	OEO	Carvacrol	Imipenem	OEO/Imipenem	Carvacrol/Imipenem
*A. baumannii* axenic	1.2	0.6	5 × 10^−4^	0.06/2.35 × 10^−5^	0.18/1.41 × 10^−4^
*P. aeruginosa* axenic	2.7	0.6	9 × 10^−4^	0.48/1.68 × 10^−4^	0.03/8.5 × 10^−5^
Co-culture	3	6	2.5 × 10^−4^	0.6/9.4 × 10^−5^	1.12/9.4 × 10^−5^

Number of replicates > 3. OEO = essential oil of *L. graveolens*.

**Table 4:** In silico molecular interactions between OXA-51 and IMP-1 enzymes with imipenem, carvacrol and thymol, in a competitive landscape (obtained through Chimera 1.16 and Discovery Studio 2021).

Enzyme	Molecule	Affinity Energy (Kcal/mol)	Interactions	Amino acids
OXA-51	Imipenem	−6.1	- Conventional hydrogen bond - Hydrogen–carbon bond - Pi-Sulfur - Pi-Alkyl	- Arg 260 * - Ser 218 - Gly 219 - Ser 80 * - Phe 111 * - Trp 114 * - Trp 222
	Carvacrol	−5.5	- Conventional hydrogen bond - Pi-Sigma - Pi-Pi T-shaped	- Arg 260 * - Phe 111 *
	Thymol	−5.4	- Pi-Pi stacked - Pi-Alkyl	- Phe 111 * - Trp 220 - Trp 114 * - Tep 222
IMP-1	Imipenem	−5.5	- Conventional hydrogen bond - Hydrogen–carbon bond - Alkyl - Pi-Alkyl	- Asp 81 - Asn 167 * - His 139 * - Val 25 * - Val 31 * - His197
	Carvacrol	−4.9	- Conventional hydrogen bond - Pi-Donor hydrogen bond - Pi-Sigma - Pi-Sulfur - Pi-Alkyl	- Asp 81 - His 139 * - Asn 167 * - His 197 - His 79 * - Cys 158
	Thymol	−4.8	- Conventional hydrogen bond - Pi-Donor hydrogen bond - Unfavorable Donor–Donor - Pi-Sigma - Pi-Alkyl	- His 77 * - Asn 167 * - His 197 - His 79 * - His 139 *

* Amino acids mostly related to the active site of OXA-51 and IMP-1 enzymes or more actively involved. They are also indicated by underlining.

## Data Availability

Data from the study are available as Supplementary Material, and at https://ciad.repositorioinstitucional.mx/jspui/ (accessed on 12 March 2024).

## Supplementary Materials

The following supporting information can be downloaded at: https://www.mdpi.com/article/10.3390/antibiotics13050444/s1, Figure S1: Macroscopic morphology of *P. aeruginosa* and *A. baumannii* colonies in co-culture ratio 1:1000. (A) Particular colony of *P. aeruginosa* with circular, punctate, small and whitish morphology. (B) Particular colony of *A. baumannii* with circular, creamy, large and whitish morphology. (C) Growing patterns in different ratios of *A. baumannii* and *P. aeruginosa*; Figure S2: Dynamic interaction of imipenem and OXA-51 (4ZDX) enzyme over time (in nanoseconds). (a) 0 ns, (b) 3 ns, (c) 10 ns, (d) 20 ns, (e) 40 ns, (f) 50 ns; Figure S3: Dynamic interaction of carvacrol and OXA-51 (4ZDX) enzyme over time (in nanoseconds). (a) 0 ns, (b) 15 ns, (c) 30 ns, (d) 40 ns, (e) 50 ns; Figure S4: Dynamic interaction of thymol and OXA-51 (4ZDX) enzyme over time (in nanoseconds). (a) 0 ns, (b) 5 ns, (c) 25 ns, (d) 34 ns, (e) 40 ns, and (f) 50 ns; Figure S5: Dynamic interaction of imipenem and IMP-1 (1DDK) enzyme over time (in nanoseconds). (a) 0 ns, (b) 2 ns, (c) 5 ns, (d) 10 ns, (e) 15 ns, (f) 25 ns, (g) 45 ns, (h) 50 ns; Figure S6: Dynamic interaction of carvacrol and IMP-1 (1DDK) enzyme over time (in nanoseconds). (a) 0 ns, (b) 5 ns, (c) 10 ns, (d) 19 ns, (e) 21 ns, (f) 25 ns, (g) 47 ns, (h) 50 ns; Figure S7: Dynamic interaction of thymol and IMP-1 (1DDK) enzyme over time (in nanoseconds). (a) 0 ns, (b) 10 ns, (c) 20 ns, (d) 30 ns, (e) 40 ns, (f) 50 ns; Table S1: Active site coordinates of the OXA-51 (PDB: 4ZDX) and IMP-1 (PDB: 1DDK) enzymes prepared in UCSF Chimera software version 1.16. The amino acids present in each region are included.
