# Supplementary material for: Lippia graveolens Essential Oil to Enhance the Effect of Imipenem against Axenic and Co-Cultures of Pseudomonas aeruginosa and Acinetobacter baumannii
Source: Antibiotics (Basel). 2024 May 14;13(5):444. doi: 10.3390/antibiotics13050444 (PMC11117758; doi:10.3390/antibiotics13050444)
Supplement: Supplementary file 1 [file antibiotics-13-00444-s001.zip › antibiotics-3008732-supplementary.pdf]

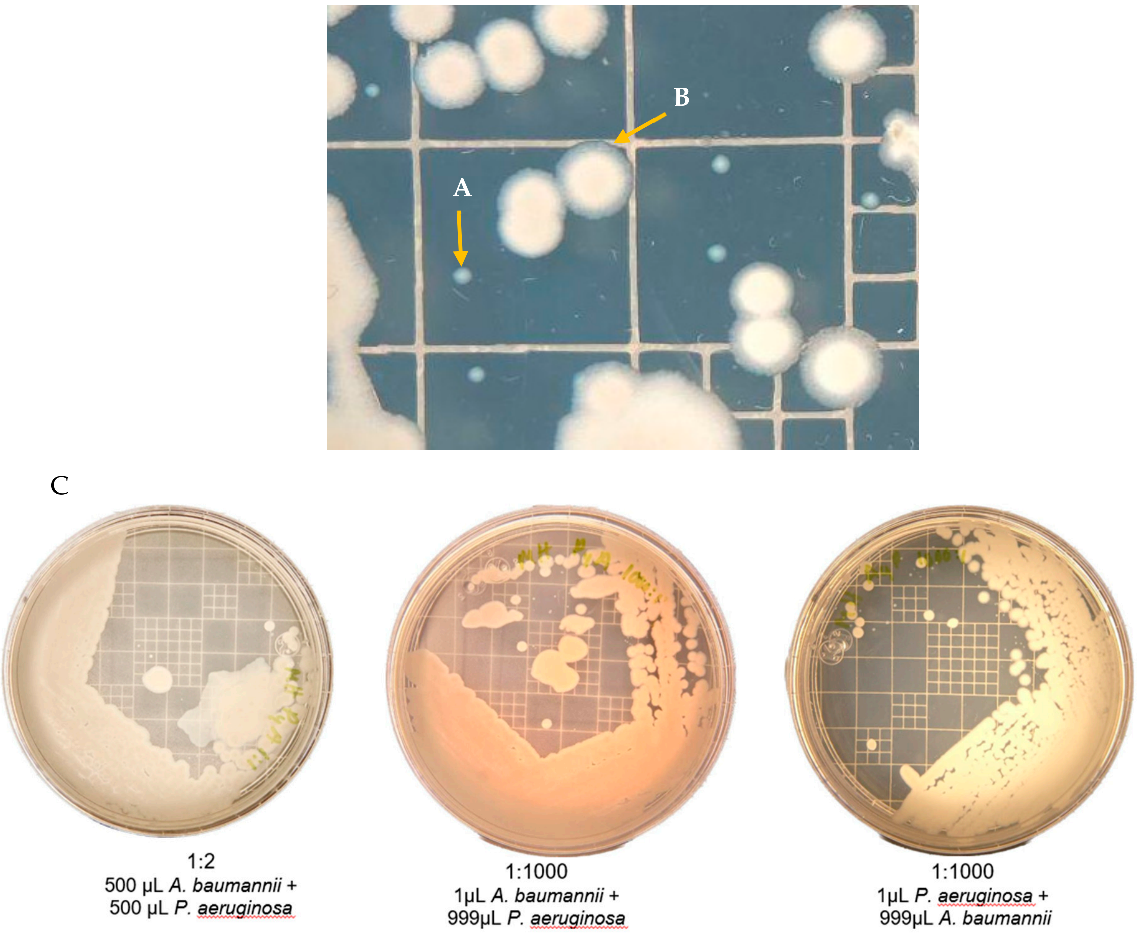

Figure S1. Macroscopic morphology of *P. aeruginosa* and *A. baumannii* colonies in co-culture ratio 1:1000. A) Particular colony of *P. aeruginosa* with circular, punctate, small and whitish morphology. B) Particular colony of *A. baumannii* with circular, creamy, large and whitish morphology. C) Growing patterns in different ratios of *A. baumannii* and *P. aeruginosa.*

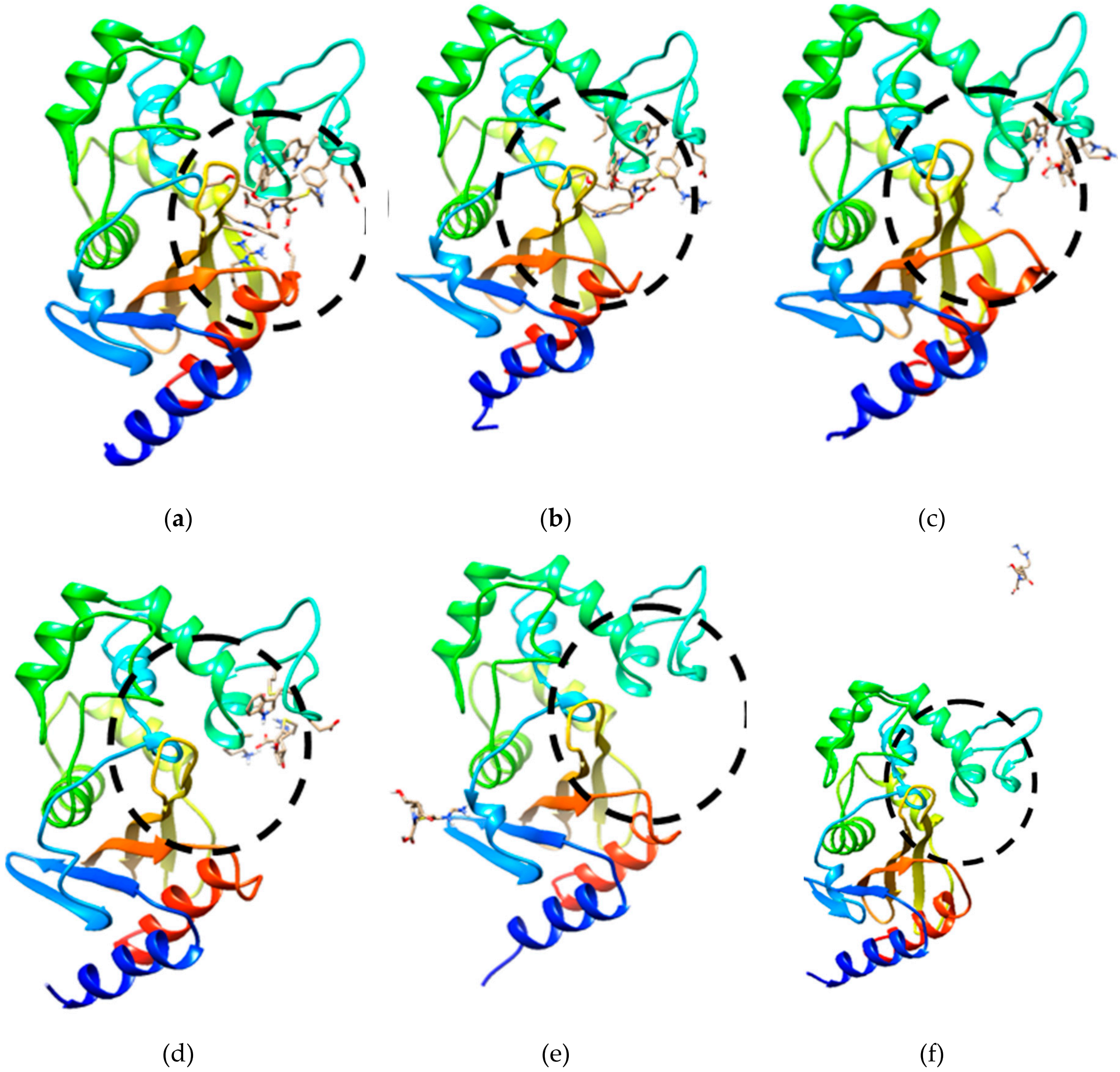

Figure S2. Dynamic interaction of Imipenem and OXA-51 (4ZDX) enzyme over time (in nanoseconds). a) 0 ns, b) 3 ns, c) 10 ns, d) 20 ns, e) 40 ns, f) 50 ns.

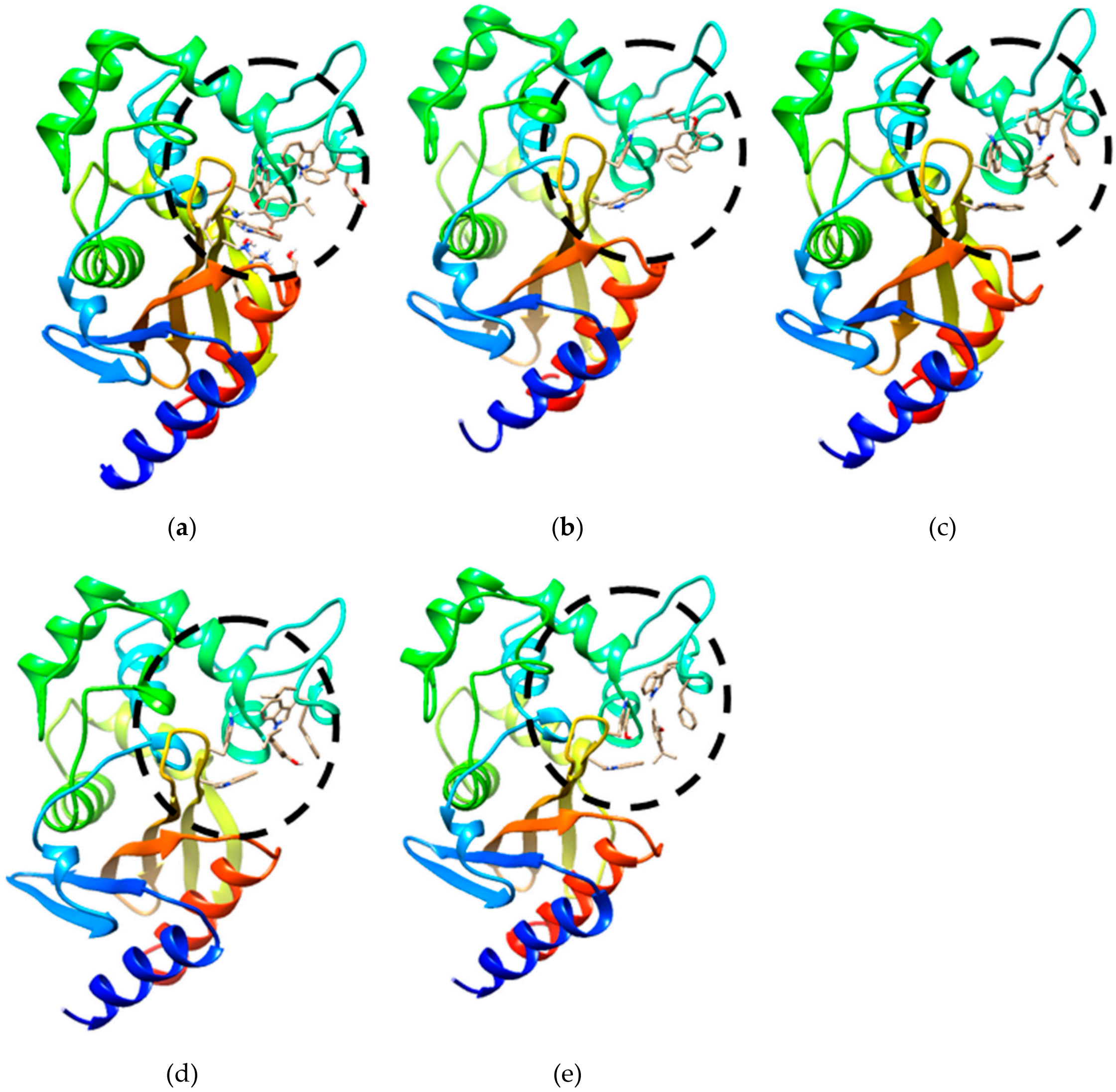

Figure S3. Dynamic interaction of carvacrol and OXA-51 (4ZDX) enzyme over time (in nanoseconds). a) 0 ns, b) 15 ns, c) 30 ns, d) 40 ns, e) 50 ns.

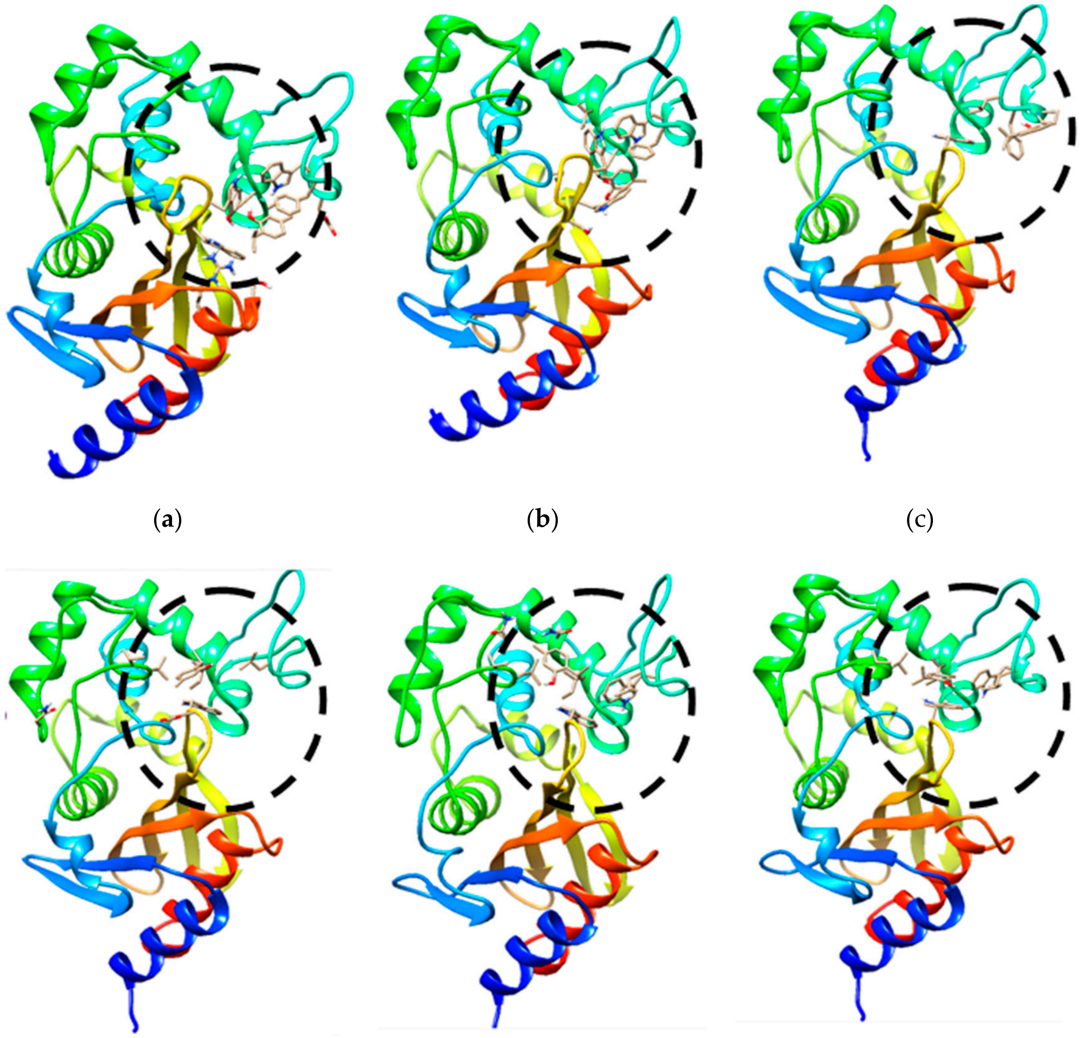

(**a**) (**b**) (c)

(d) (e) (f)

Figure S4. Dynamic interaction of thymol and OXA-51 (4ZDX) enzyme over time (in nanoseconds). a) 0 ns, b) 5 ns, c) 25 ns, d) 34 ns, e) 40 ns, and f) 50 ns.

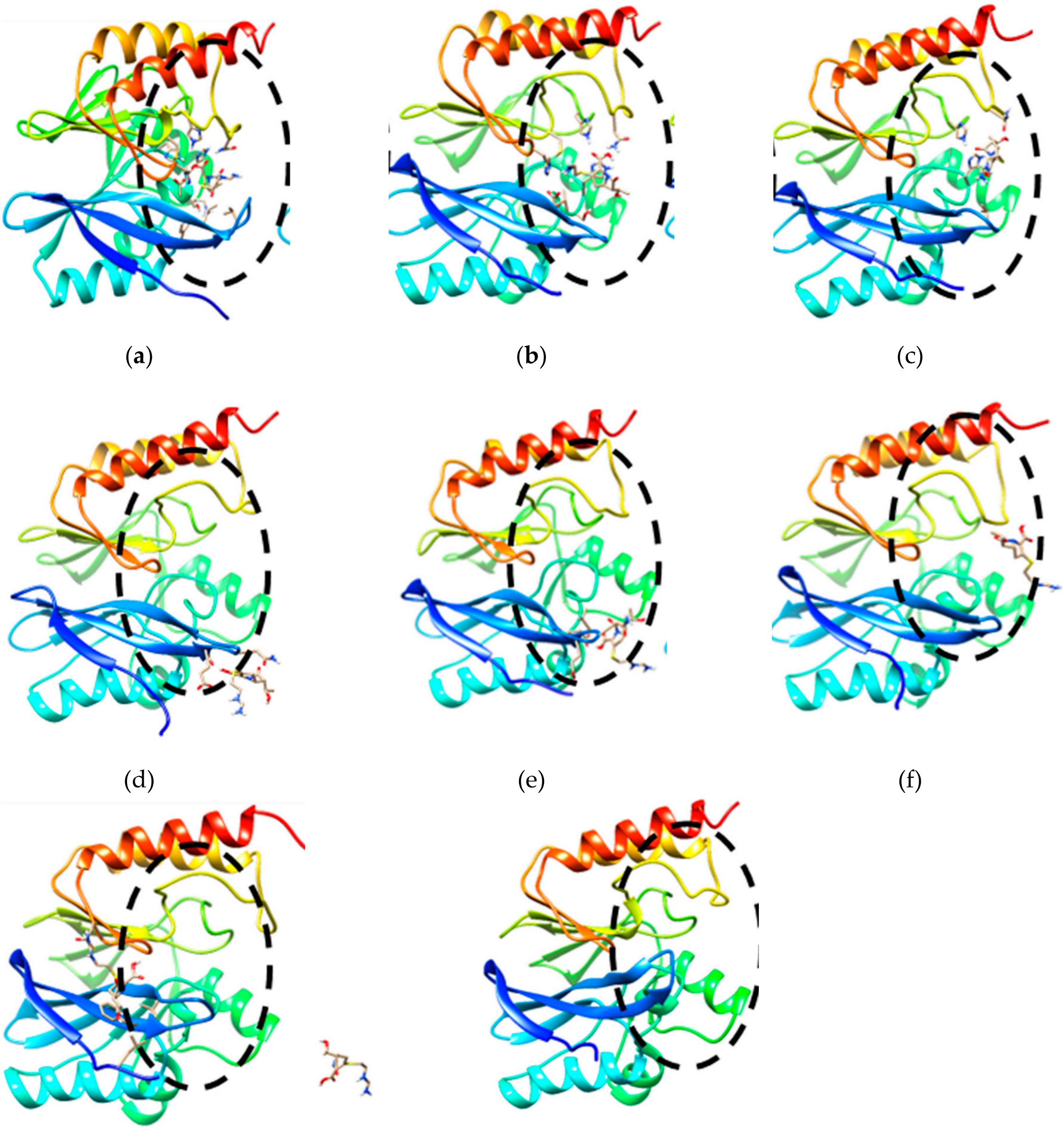

Figure S5. Dynamic interaction of imipenem and IMP-1 (1DDK) enzyme over time (in nanoseconds). a) 0 ns, b) 2 ns, c) 5 ns, d) 10 ns, e) 15 ns, f) 25 ns, g) 45 ns, h) 50 ns.

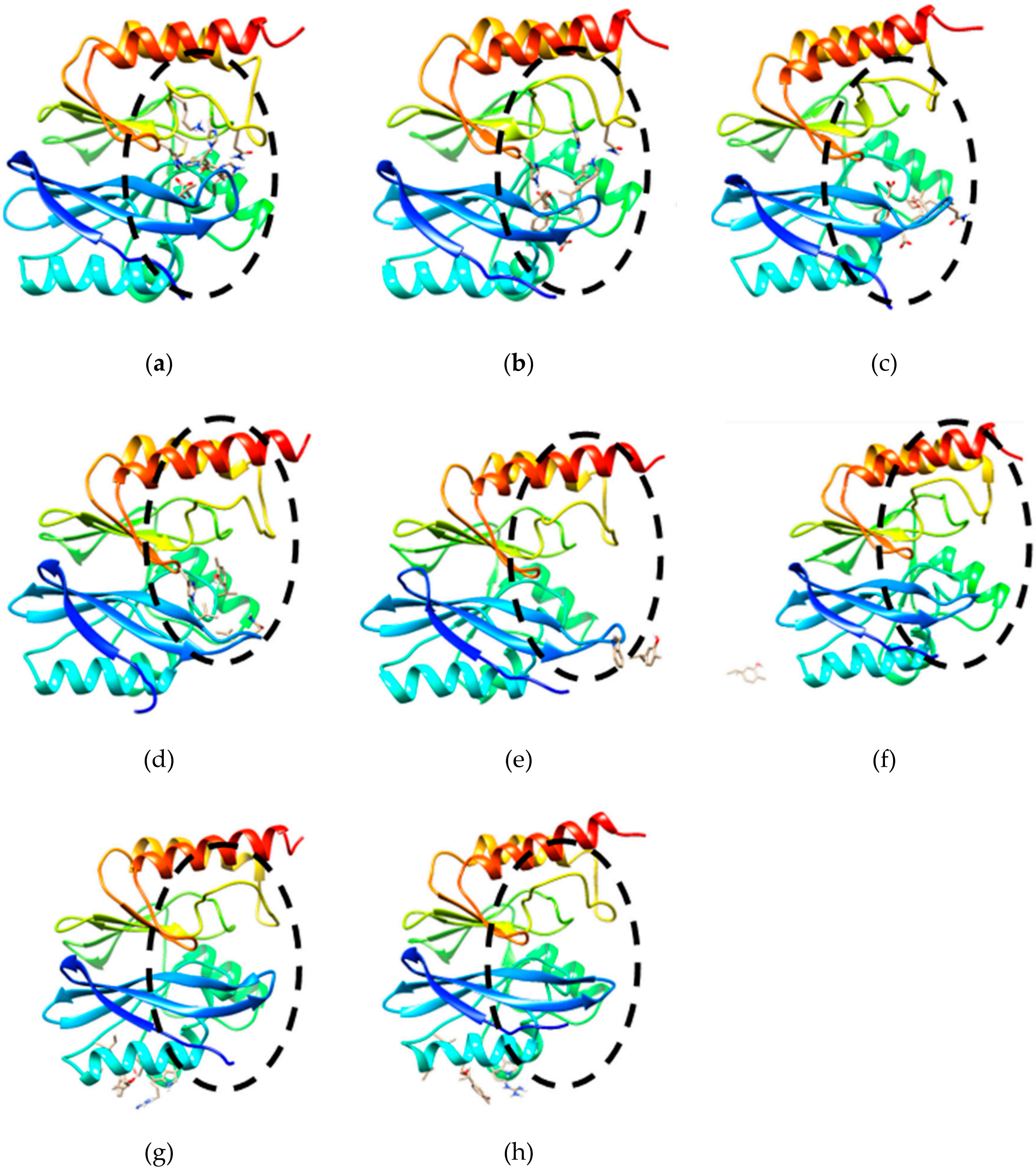

Figure S6. Dynamic interaction of carvacrol and IMP-1 (1DDK) enzyme over time (in nanoseconds). a) 0 ns, b) 5 ns, c) 10 ns, d) 19 ns, e) 21 ns, f) 25 ns, g) 47 ns, h) 50 ns.

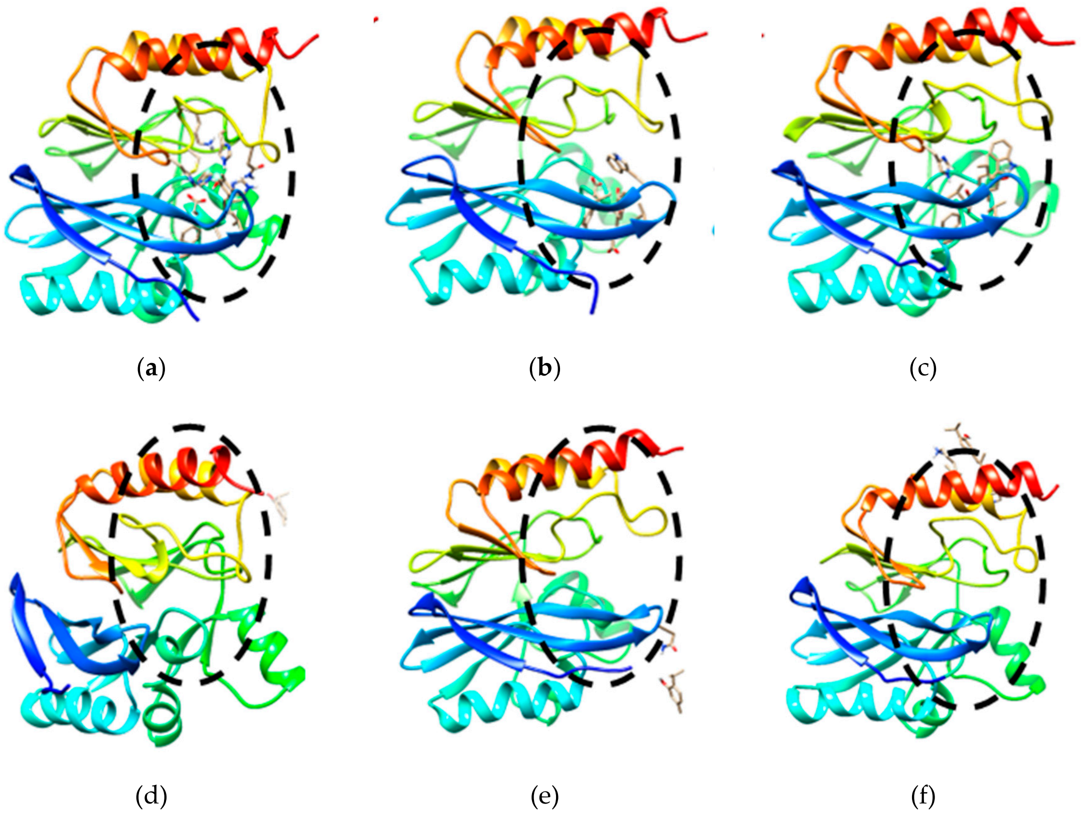

Figure S7. Dynamic interaction of thymol and IMP-1 (1DDK) enzyme over time (in nanoseconds). a) 0 ns, b) 10 ns, c) 20 ns, d) 30 ns, e) 40 ns, f) 50 ns.

Table S1. Active site coordinates of the OXA-51 (PDB: 4ZDX) and IMP-1 (PDB: 1DDK) enzymes prepared in UCSF Chimera software version 1.16. The amino acids present in each region are included.

Enzyme	Center	Size	Aminoacids
OXA-51	23.1202 (x)<br>30.4202 (y)<br>21.6143 (z)	28.5741 (x)<br>30.8404 (y)<br>37.1683 (z)	Lys83, Ser127, Trp166, Lys217, Arg260, Ser80, Phe111, Trp114, Ile129, Leu167, Ser218, Trp220, Trp222
IMP-1	11.997 (x)<br>4.0829 (y)<br>57.6211 (z)	26.9028 (x)<br>20.7375 (y)<br>15.905 (z)	Glu23, Val25, Trp28, Phe51, Asp81, His79, His197, His77, Cys158, His139, Lys161
